# People's wellbeing, civic capital and sustainable practices: Evidence from the European Values Study survey

**DOI:** 10.3389/fsoc.2022.1048397

**Published:** 2023-01-04

**Authors:** Marco Ciziceno

**Affiliations:** Department of Economics, Business and Statistics, University of Palermo, Palermo, Italy

**Keywords:** sociology, environmentalism, wellbeing, social capital, civicness

## Abstract

The climate change issue is showing an unprecedented level of awareness in the political realm. Changing occasional sustainable practices into stable behaviors is the challenge that policymakers face. However, what makes people environmentally aware is an unsolved question, and research on this direction is in evolution. This paper examines factors that promote environmentally responsible behaviors. The study tests the hypothesis that people's wellbeing (SWB) predisposes individuals toward environmentalism. The mechanism of social and civic capital may underlie this association since people reporting higher wellbeing levels show empathy, solidarity, and greater civic engagement. This hypothesis is examined in the context of the European Union using micro-data from the European Values Study—EVS (wave 2017–2022). Results support the hypothesis that people's life satisfaction is compatible with the environmental mindset, given that those who report higher wellbeing express civicness and share pro-environmental beliefs and values. Evidence from this research suggests that supporting SWB growth may offer a fertile ground for promoting ecological awareness and developing more sustainable societies.

## 1. Introduction

In recent times, environmental issues have dominated political agendas worldwide, and many spontaneous movements claim urgent actions for a more sustainable and inclusive society^1^ (Signoretta et al., 2020). At the individual level, adopting eco-friendly lifestyles is essential to coping with ecological concerns (York et al., 2003). For example, reducing household energy use, supporting the recycling principle, and limiting food waste in favor of greener consumption are practical solutions that individuals can do to limit their ecological footprint (Dietz et al., 2007; Thompson et al., 2011).

However, a better understanding of environmental attitudes is the prerequisite for effectively transforming people's lifestyles. The first step is identifying factors that encourage or discourage such attitudes. Literature indicates many drivers of environmental intentions, such as social and personal norms (Schwartz, 1994, 1996; Stern et al., 1999; Turaga et al., 2010), awareness of consequences (Gifford and Nilsson, 2014), and locus of control (Coşkun et al., 2022). Studies also indicate that altruism and social engagement are relevant factors for pro-environmentalism (Dietz et al., 2002; Knez, 2016). Based on this argument, Schultz (2000, 2001) has found that sustainably oriented people support each other in a social-altruistic manner, whereas Macias (2016) has found that social capital variables are good predictors of sustainable practices. It means that social and civic capital are essential for pro-environmental behaviors, given the importance of citizen cooperation in preserving the environment (see Andreoni, 1990; Steg and De Groot, 2010; Goldy and Piff, 2020).

From another perspective, growing studies point out that more satisfied people often operate in a cooperative and trustful manner (Tov and Diener, 2009). For example, Kushlev et al. (2020) have found that both happiness and life satisfaction positively affect prosocial behaviors, whereas recently, Ciziceno and Pizzuto (2022) argue that satisfied people accept moral and civic values and less justify the extra-legal ones.

Although these findings suggest that people's subjective wellbeing (SWB) elicits civicness and that civicness explains, in part, environmental behavior, few studies have linked SWB directly to people's ecological orientation.

This paper extends the current research on ecocentrism vs. anthropocentrism (see Thompson and Barton, 1994) and subjective wellbeing (SWB). The idea is that higher SWB is associated with individuals' ecocentric orientation (than anthropocentric one). Being more satisfied with their own lives makes people sensitive to their community's health and wellbeing (in other words, helping them to be more civic). The relationship between environmentalism and SWB is explored using micro-data from the European Values Study (EVS) wave 2017–2022. The results suggest that life satisfaction is positively associated with ecocentrism, and this association is stable after several controls. To further confirm the hypothesis that SWB activates a virtuous cycle encompassing both sustainable and civic attitudes, it has been tested the association between life satisfaction and a set of variables proxy for individuals' civicness. The main finding from this exercise is that people with higher life satisfaction systematically reject those behaviors at the expense of others or their community, such as falsely claiming benefits or accepting a bribe.

This paper contributes to the literature on ecological orientation and subjective wellbeing from a different two-fold perspective. Firstly, it adds further knowledge to the positive factors underlying environmental orientations, namely peoples' wellbeing. Secondly, it supports studies that have found as civic duty helps urban development and increases environmentalism (Bamberg and Möser, 2007; Corral-Verdugo et al., 2009). Finally, the policy implications of such findings are discussed.

## 2. Literature review

### 2.1. Environmental orientation: Ecocentrism vs. anthropocentrism

Environmental orientation encompasses a series of individual attitudes aimed at protecting both the physical and the social environment (Axelrod and Lehman, 1993; Dietz et al., 2007; Prati et al., 2017). However, a crucial point in environmental studies is the relationship between peoples' orientation and their actual behavior. Mainstream literature reports that people with a pro-environmental orientation act environmentally consequently (Schlegelmilch et al., 1996; Kollmuss and Agyeman, 2002). Kopnina (2012, p. 611) argues that people with ecocentric orientations are much more likely to actually act upon their values, attitudes, and beliefs in order to protect the environment than those with anthropocentric orientations.

According to the Values Belief Norm Theory^2^ (Stern et al., 1999), individuals' values influence pro-environmental behaviors through the mediating effect of beliefs and personal norms. For example, if people believe that car use causes problems for the environment, it is more likely that they feel morally obligated to limit their personal use of the car (Hiratsuka et al., 2018). Ajzen and Fishbein (1975) have demonstrated that intentions are a predictor of volitional behavior, and cumulative results (see Hinesa et al., 1987; Sheppard et al., 1988) indicate the predictive utility that attitudes, values, and social norms have on human behaviors, including pro-environment actions^3^. However, environmental beliefs do not always translate into ecological efforts, and this gap is known as the knowledge-concern-action paradox (Lenzen and Cummins, 2011).

Besides, according to Schultz (2000, 2001), people's environmental concerns about their health or other people are a reliable proxy of environmentalism. Schultz (2000, 2001), adopting the tripartite structure of egoistic, socio-altruistic, and biospheric people's orientation, has found that those who meet an egoistic orientation tend to emphasize the consequences of environmental deterioration only for themselves, whereas those who express apprehension for other humans have a socio-altruistic' view. Lastly, individuals representing a biospheric orientation stress the consequences of environmental deterioration for humans and non-humans (including animals, plants, and ecosystems). Starting from the former perspective, Thompson and Barton (1994) have classified environmental orientation using the ecocentric, anthropocentric, and apathy scales. In this approach, Schultz's egoistic and socio-altruistic dimensions collapse into a single construct indicating an ecocentric orientation. Individuals with an ecocentric orientation are more sensitive about problems afflicting nature. For this reason, the literature argues that relevant intrinsic motives for pro-environmental behaviors are prosociality and altruism (Owen and Videras, 2006). On the contrary, anthropocentrism is a human-centered approach (Abun and Racoma, 2017). In this view, nature essentially exists to meet human needs, and the main reason for preserving biodiversity is to ensure people live. People who express environmental apathy consider the risks related to the environment as exaggerated (Yildiz and Erciş, 2022) and tend to minimize the relevance of ecological concerns.

### 2.2 The relationship between environmental orientation and subjective wellbeing

During the last few years, literature on subjective wellbeing (SWB) has grown dramatically as an intersection between sociology, psychology, and economics to study people's evaluation of their lives (Kroll, 2014). This research area has become popular in the political arena, even if sociologists do not consider SWB a big topic (Veenhoven, 2000). However, scholars (Nolan and Lenski, 2004; Kroll, 2014) agree that wellbeing study addresses crucial questions for sociology, connecting the individual-level with the social one. The SWB is the combination of both individuals' emotional states (i.e., pleasant and unpleasant emotions) and cognitive evaluation of his/her life (i.e., life satisfaction, see Diener et al., 1985). Experimental studies have demonstrated that life satisfaction is a separable component of SWB that could be analyzed independently (Diener et al., 2002). Besides, life satisfaction refers to a general evaluation of individuals' lives, and research on this topic often relies on life satisfaction as a proxy of SWB. Indeed, compared to other components of SWB (e.g., happiness), Fujita and Diener (2005) have found that life satisfaction is less susceptible to cultural differences and that it shows appreciable psychometric properties.

Existing literature indicates an association between SWB and pro-environmental behaviors, even if part of such research offers contrasting or less generalizable results. For example, Brown and Kasser (2005) have found that SWB and ecologically responsible behaviors are compatible, demonstrating that people with intrinsic value orientation are both happier and more ecological. However, they provide results from two small-size samples mainly composed of students and voluntary respondents. Similarly, Prati et al. (2017), using a restricted sample of students, tested the causal relationship between social wellbeing and pro-environmental behavior. They show that a reciprocal relationship exists, in which acting pro-environmentally increases SWB and *vice versa*. Suárez-Varela et al. (2016) have found that acting environmentally has either a positive or no significant influence on SWB. Other studies, such as those of Venhoeven et al. (2013), indicate that eco-friendly behaviors increase SWB, but only partially (i.e., they affect eudaimonic wellbeing). Welsch and Kühling (2010) have demonstrated that life satisfaction is positively associated with environmental-friendly behaviors (e.g., green consumption), and such association may be driven by peoples' intrinsic motives such as empathy or altruism.

However, despite their ecological awareness, people do not ever operate environmentally (Lenzen and Cummins, 2011). Furthermore, greener solutions are often more expensive than traditional ones, and this may discourage any complaint intentions. Other studies have focused on the negative influences environmental concerns have on SWB (Cottrell, 2003). For example, Rehdanz and Maddison (2008) find that concern about noise levels and air pollution reduces SWB, whereas Ferrer-i-Carbonell and Gowdy (2007) indicate that caring about climate change factors (i.e., ozone layer) constitutes a mental stressor for individuals' wellbeing.

## 3. Data and measures

### 3.1 Data description

Data used in this study are drawn from the European Values Study (EVS 2017–2022)^4^. The EVS survey collects large-scale, cross-national information about a multitude of social, political, and cultural issues of European citizens. The latest wave available (2017–2022) also includes detailed information on public opinion about the actual environmental situation. The survey data were collected by representative single-stage or multi-stage sampling of the adult population (18 years old and over). The EVS dataset is fully exploited, and the list of countries included in the analysis has been determined by data availability.

### 3.2 Measures

This subsection describes all the survey items included in the analyses.

Ecocentric vs. Anthropocentric attitudes: a 3-item scale measures respondents' environmental orientation. The items asked the following questions: (1) It is just too difficult for someone like me to do much about the environment, (2) There are more important things to do in life than protect the environment, and (3) Many of the claims about environmental threats are exaggerated.

Participants' answers range from 1 (strongly agree) to 5 (strongly disagree), where higher scores (strongly disagree) indicate their ecocentric orientation. A single-item using a scenario asks the following question: here are two statements people sometimes make when discussing the environment and economic growth. Which of them comes closer to your own point of view? Respondents may answer by giving priority to the environment (ecocentric orientation) or preferring economic growth and job creation (anthropocentric orientation). Factor analysis tests whether all items used are loaded on the same factor. The study uses principal components as a method of extraction (varimax rotation) and indicates that all the items led to a one-factor solution (range factor loadings 0.65 to 0.77), explaining 50.01% of the variance.

Life satisfaction: a single-item measures the satisfaction with life. Participants in the EVS answered the following question: all things considered, how satisfied are you with your life as a whole these days? The score ranges from 1 (completely dissatisfied) to 10 (completely satisfied).

Control variables: literature indicates numerous demographic and socio-economic factors shaping environmental attitudes, such as education (Kopnina, 2012), age, and economic status (for a review, see Gifford and Nilsson, 2014). The control variables included in the analyses are gender (0 = female; 1 = male), the age of respondents (recoded as 1 = 15–29 years; 2 = 30–49 years; 3 = 50 and more years), educational level (ISCED classification harmonized in 8 categories from 1 = uncomplete elementary education to 8 = university degree), household total net income^5^ (recoded into three levels as 1 = low; 2 = middle and 3 = high), marital status (recoded into the following categories: married; registered partnership; widowed; divorced; separated; never married and never registered partnership). The analyses also include individuals' per-capita GDP^6^ as a control variable for life satisfaction levels across countries. The SWB literature stresses the notion that happiness parallel increases to personal income and economic growth. However, Easterlin (1995) has found that turning the satiation point, such a relationship loses statistical significance. To control for the effect of the implementation of sustainable policies on individuals' environmental attitudes, the country-specific score of the Sustainable Development Goal index (SDGi)^7^ has been added to the models.

Robustness check variables: the EVS survey uses multiple response questions measuring individuals' civicness, such as their beliefs on civic duty, community respect, and fairness. Those variables are included in the analyses as a robustness check (see subsection 5.1). The 4-items measure respondents' justification of different non-compliant behaviors: (1) falsely claiming government benefits; (2) cheating on tax; (3) someone accepting a bribe; (4) avoiding a fare on public transport. Respondents' answers range from 1 (never justified) to 10 (always justified). To facilitate the interpretation of the results, the items have been recoded, and the answer is never justified… assumes the highest value (=10), indicating better compliance with social norms and higher prosocial orientation. The factor analysis indicates that all the items used led to a one-factor solution (range factor loadings 0.77 to 0.73), explaining 57.01% of the variance.

## 4. Analytical strategy and method

This paper aims to investigate the role of life satisfaction as a potential driver for individuals' environmental orientation. Thus, the research hypotheses are the following:

H1: higher life satisfaction (LS) is associated with ecocentric orientation than with anthropocentric one.
H2: this association is robust after controlling for both individuals' socio-demographic characteristics and country-specific variables.
H3: life satisfaction is also linked to other relevant civic and ethical beliefs (under the assumption that life satisfaction encourages both pro-environmental and civic values).

To investigate the relationship between ecocentric orientation and life satisfaction, it has been used an ordered logit model. The coefficients are estimated using maximum likelihood (MLE). The ordered logit models are discrete choice models used in several econometric applications, including, for example, individuals' recycling preferences (Nixon et al., 2009). The model considers the ordinality of response variables and estimates the category that most closely fits with respondents' feelings on questions^8^ (Grilli and Rampichini, 2014). The ordinal logistic regression model used is based on the following specification:


(1) $$\begin{array}{l} y_{i}^{*}=\alpha +\beta LS_{i}+{{\gamma^{\prime }x}_{i}+\varepsilon }_{i} \end{array}$$

where, *x*_*i*_ is a matrix of variables indicating the socio-economic and demographic individuals' characteristics (such as age, gender, and educational level) and other control variables often associated with environmental behavior, such as per-capita GDP and the SGDi score. LS_i_ is the reported level of life satisfaction for the i-th individual participating in the survey. Then, α is the intercept term, and ε_*i*_ is the error term capturing the unobserved individuals' characteristics. Finally, $y_{i}^{*}$is the dependent variable indicating the ecocentrism level for the i-th individual, that in this dataset can take values from 1 to 5. To eases the interpretation of results for each explanatory variable (except life satisfaction^9^ and continuous variables), one level has been omitted. The combination of omitted levels gives the characteristics of the reference group, and the estimate for this group is reflected by the coefficient for the constant. Finally, Equation (1) is augmented with a set of variables indicating prosocial orientation, to shed light on hypothesis 3.

## 5. Results

Table 1 reports the baseline results from Equation (1) estimation of the whole sample. The ordered log-odds models in Table 1 indicate that life satisfaction is associated with different measures of ecocentrism (H1). Notably, for one unit of increase in the self-reported level of life satisfaction (from 1 to 10 points), the likelihood of environmental verbal commitment increases by 13% (odds ratio = 1.133, SE = 0.012, see Table 1, model 1). A potential explanation of such a relationship may reside in the fact that satisfied people are more socially responsible and sensitive to the community's needs than unsatisfied ones (Tov and Diener, 2009). This, in turn, may drive their attitudes toward altruistic social practices, which also include environmentalism. Similar evidence has been found by Welsch and Kühling (2010), who showed as life satisfaction increases environmental friendliness and pro-environmental behaviors. Kasser (2017) has provided empirical evidence that a positive correlation between wellbeing and pro-ecological behaviors exists.

**Table 1:** Baseline estimations.

	**Model** ** (1)**	**Model** ** (2)**	**Model** ** (3)**	**Model** ^a^ ** (4)**
	**Environmental verbal commitment**	**Environmental importance**	**Environmental awareness**	**Protecting environment vs. economic growth**
**Independent variable**				
Life satisfaction	1.133^***^	1.086^***^	1.052^***^	1.061^***^
	(0.012)	(0.012)	(0.011)	(0.015)
**Age of respondent (15–29 years** = **reference group) and Gender (dummy variable)**				
30–49 years	1.173^***^	1.245^***^	1.122^**^	1.231^***^
	(0.049)	(0.052)	(0.053)	(0.071)
50 and over years	1.151^**^	1.472^***^	1.136^**^	1.430^***^
	(0.081)	(0.089)	(0.067)	(0.100)
Gender (1 = male)	1.174^***^	1.280^***^	1.328^***^	1.102^***^
	(0.041)	(0.040)	(0.035)	(0.038)
**Educational level attained (incomplete elementary** = **reference group)**				
Completed elementary	0.948	1.329	1.003	1.138
	(0.129)	(0.262)	(0.220)	(0.160)
Incomplete secondary/technical type	1.255	1.270	1.182	1.401^***^
	(0.195)	(0.250)	(0.234)	(0.183)
Complete secondary/technical type	1.112	1.173	1.057	1.452^***^
	(0.175)	(0.250)	(0.244)	(0.200)
Incomplete secondary/university-preparatory type	2.360^***^	2.730^***^	2.146^***^	1.970^***^
	(0.379)	(0.674)	(0.506)	(0.325)
Complete secondary/university-preparatory type	1.369	1.257	1.253	1.538^***^
	(0.271)	(0.259)	(0.257)	(0.240)
University without degree	1.961***	1.747^***^	1.818^***^	2.079^***^
	(0.350)	(0.360)	(0.402)	(0.361)
University with degree	2.252^***^	1.833^***^	2.095^***^	2.454^***^
	(0.426)	(0.372)	(0.449)	(0.434)
**Marital status (never married** = **reference group)**				
Married	0.746^***^	0.713^***^	0.730^***^	0.731^***^
	(0.043)	(0.043)	(0.032)	(0.039)
Registered partnership	1.024	0.942	1.133	1.081
	(0.110)	(0.108)	(0.114)	(0.109)
Widowed	0.572^***^	0.602^***^	0.657^***^	0.639^***^
	(0.040)	(0.046)	(0.038)	(0.040)
Divorced	0.878^**^	0.854^***^	0.851^***^	0.810^***^
	(0.047)	(0.050)	(0.052)	(0.056)
Separated	1.055	1.127	1.120	1.020
	(0.105)	(0.137)	(0.095)	(0.109)
**Household total net income (low** = **reference group)**				
Middle	1.135^***^	1.070^**^	1.038	1.073^*^
	(0.026)	(0.034)	(0.035)	(0.040)
High	1.354***	1.184^***^	1.154^**^	1.170^***^
	(0.060)	(0.056)	(0.067)	(0.063)
Observations	48,906	48,949	47,681	44,599

p-value = ^***^p < 0.01, ^**^p < 0.05, ^*^p < 0.1.

Standard errors are reported in parentheses.

a Given the binary response of the item, the logistic regression model it has been used as method of estimation.

The set variables included in the models as covariates indicate that age and gender are predictors of ecocentrism. Consistently to existing studies, aged people tend to be more interested in the environmental issue than younger ones, whereas being female increases the log odds of being environmentally responsible by 32% more than male (see Table 1, model 3). In support of these results, Casey and Scott (2006) have found that younger people are less ecocentric than older, whereas Scannell and Gifford (2013) pointed out that women are more environmentally concerned than men, and for this reason, they frequently act environmentally.

Looking at socio-demographic variables, having a higher educational level (i.e., university degree) and being married or divorced are predictors of ecocentrism (see Table 1, models 1, 2, 3, 4). In a meta-analysis have found that both environmental knowledge and education level determine pro-environment behaviors. In line with these findings, Chanda (1999) has found that individuals higher in education display greater attention to environmental concerns. Also, results from Table 1 indicate that belonging to the upper level of income (from 1 to 3) increases the likelihood of being more ecocentric. Studies supporting this finding suggest that, for example, in Germany, environmentalists are often people from the middle and middle-upper classes rather than lower (Balderjahn, 1988).

To clearly identify the association between life satisfaction and ecocentrism, Equation (1) has been augmented with control variables often associated with SWB and environmental intentions. The results of the robustness check are reported in Table 2. Notably, after the introduction of per-capita GDP and the Sustainable Development Goal index (SDGi) results are very similar and broadly unchanged with respect to the baseline estimation (see Table 2). In line with the H2, results show that the increases in per-capita GDP are associated likelihood that individuals prefer respecting the environment (Table 2, models 1, 2, 3, 4). An explanation of such a finding has been provided by Inglehart (1997). According to the author, in economically developed countries, individuals often shift their reference values from materialistic to post-materialistic ones, including environmental protection and ecological respect.

**Table 2:** Log-odds estimations with additional control variables.

	**Model** ** (1)**	**Model** ** (2)**	**Model** ** (3)**	**Model** ** (4)**
	**Environmental verbal commitment**	**Environmental importance**	**Environmental awareness**	**Protecting environment vs. economic growth**
**Independent variable**				
Life satisfaction	1.092^***^	1.035^***^	1.015	1.027^*^
	(0.010)	(0.011)	(0.011)	(0.014)
**Age of respondent (15–29 years** = **reference group) and Gender (dummy variable)**				
30–49 years	1.065	1.124^***^	1.001	1.121^*^
	(0.048)	(0.050)	(0.055)	(0.072)
50 and over years	0.909^**^	1.190^***^	0.920	1.218^***^
	(0.041)	(0.066)	(0.053)	(0.082)
Gender (1 = male)	1.256^***^	1.349^***^	1.396^***^	1.110^**^
	(0.046)	(0.050)	(0.045)	(0.048)
**Educational level attained (incomplete elementary** = **reference group)**				
Completed elementary	1.005	1.109	0.760^*^	1.165
	(0.166)	(0.190)	(0.119)	(0.218)
Incomplete secondary/technical type	1.469^**^	1.197	0.984	1.559^***^
	(0.283)	(0.218)	(0.127)	(0.256)
Complete secondary/technical type	1.349	1.228	0.932	1.629^***^
	(0.256)	(0.233)	(0.153)	(0.263)
Incomplete secondary/university-preparatory type	2.106^***^	1.743^***^	1.352^*^	1.580^**^
	(0.381)	(0.331)	(0.229)	(0.289)
Complete secondary/university-preparatory type	2.019^***^	1.531^**^	1.232	2.104^***^
	(0.389)	(0.299)	(0.195)	(0.393)
University without degree	2.413^***^	1.680^***^	1.631^***^	2.612^***^
	(0.458)	(0.309)	(0.264)	(0.457)
University with degree	3.041^***^	1.794^***^	1.994^***^	3.249^***^
	(0.581)	(0.356)	(0.332)	(0.601)
**Marital status (never married** = **reference group)**				
Married	0.951	0.896^**^	0.861^***^	0.842^***^
	(0.029)	(0.042)	(0.037)	(0.037)
Registered partnership	0.967	0.880	1.093	1.033
	(0.081)	(0.091)	(0.087)	(0.076)
Widowed	0.732^***^	0.809^***^	0.783^***^	0.781^***^
	(0.032)	(0.045)	(0.038)	(0.040)
Divorced	0.993	0.959	0.939	0.887^*^
	(0.042)	(0.047)	(0.051)	(0.056)
Separated	1.131	1.154	1.063	0.963
	(0.091)	(0.103)	(0.100)	(0.094)
**Household total net income (low** = **reference group)**				
Middle	1.151^***^	1.065	1.034	1.087^**^
	(0.032)	(0.041)	(0.036)	(0.046)
High	1.300^***^	1.146^***^	1.124^**^	1.177^***^
	(0.070)	(0.060)	(0.061)	(0.069)
**Additional control variables**				
Log per-capita GDP	1.376^***^	1.858^***^	1.574^***^	1.714^***^
	(0.113)	(0.216)	(0.143)	(0.195)
SGD_index	1.006	0.987	0.988	0.989
	(0.013)	(0.011)	(0.011)	(0.014)
Observations	37,820	37,833	37,144	34,486

p-value= ^***^p < 0.01, ^**^p < 0.05, ^*^p < 0.1.

Standard errors are reported in parentheses.

### 5.1 Robustness check: The relationship between SWB and civicness

Equation (1) has been re-estimated by adding a set of civic norms as response variables and life satisfaction as a predictor (see Table 3). Respect for civic norms is an expression of social capital and helps people live in a more stable and predictable environment (Putnam, 2000; Bjørnskov, 2003). Studies have shown as following these norms prevents natural resources degradation and limit individuals' opportunistic behaviors (Pretty and Ward, 2001; Owen and Videras, 2006). Other empirical findings link the effects of SWB on social and civic capital (see Bjørnskov, 2003; Helliwell and Putnam, 2004; Kroll, 2011). To further confirm the hypothesis that life satisfaction is a predictor (or driver) of positive outcomes not limited to environmental issues, I check for the effect of life satisfaction on a set of other variables expressing civicness (H3). Civic behavior often refers to actions aimed at limiting free riding for the benefit of others or the community. Examples of such behaviors include donating blood and organs and volunteerism (Putnam, 1993), but also compiling with taxes and respecting the physical environment. This definition of civicness drowns back from the socialization theory (Durkheim, 1951[1897]; Parsons, 1967), which solves the puzzle of civic engagement by looking at the individuals' level of internalization of norms or other institutional factors such as the social cohesion within a society.

**Table 3:** Life satisfaction and civic capital.

	**Model** ** (1)**	**Model** ** (2)**	**Model** ** (3)**	**Model** ** (4)**
	**Claiming false state benefits (10 = never justifiable)**	**Cheating on tax (10 = never justifiable)**	**Accepting a bribe** ** (10 = never justifiable)**	**Cheating on public transport (10 = never justifiable)**
**Independent variable**				
Life satisfaction	1.096^***^	1.100^***^	1.114^***^	1.087^***^
	(0.017)	(0.017)	(0.019)	(0.014)
**Age of respondent (15–29 years** = **reference group) and Gender (dummy variable)**				
30–49 years	1.415^***^	1.216^***^	1.624^***^	1.409^***^
	(0.102)	(0.050)	(0.108)	(0.096)
50 and over years	2.150^***^	1.622^***^	2.454^***^	2.312^***^
	(0.169)	(0.097)	(0.189)	(0.197)
Gender (1 = male)	1.066^**^	1.360^***^	1.266^***^	1.049^**^
	(0.034)	(0.053)	(0.051)	(0.024)
**Educational level attained (incomplete elementary** = **reference group)**				
Completed elementary	0.901	1.117	1.060	0.964
	(0.253)	(0.146)	(0.150)	(0.156)
Incomplete secondary/technical type	1.431^*^	1.259	1.192	0.940
	(0.289)	(0.214)	(0.177)	(0.147)
Complete secondary/technical type	1.343	1.219	1.056	0.777
	(0.391)	(0.216)	(0.217)	(0.158)
Incomplete secondary/university-preparatory type	1.676^**^	1.349^*^	0.987	0.893
	(0.344)	(0.239)	(0.192)	(0.150)
Complete secondary/university-preparatory type	1.309	1.159	1.092	0.779
	(0.322)	(0.229)	(0.227)	(0.151)
University without degree	1.662^**^	1.343	1.410^*^	0.727^*^
	(0.349)	(0.242)	(0.263)	(0.127)
University with degree	1.516^**^	1.264	1.348^*^	0.675^**^
	(0.306)	(0.249)	(0.236)	(0.116)
**Marital status (never married** = **reference group)**				
Married	1.248^***^	1.158^***^	1.195^***^	1.535^***^
	(0.081)	(0.059)	(0.063)	(0.076)
Registered partnership	0.925	0.926	0.930	1.001
	(0.128)	(0.077)	(0.110)	(0.091)
Widowed	1.292^***^	1.270^***^	1.127	1.643^***^
	(0.103)	(0.102)	(0.124)	(0.143)
Divorced	1.108^**^	0.978	1.018	1.093^**^
	(0.049)	(0.056)	(0.054)	(0.048)
Separated	0.918	1.071	0.924	1.082
	(0.127)	(0.101)	(0.112)	(0.113)
**Household total net income (low** = **reference group)**				
Middle	1.065	0.989	1.024	0.907^***^
	(0.048)	(0.030)	(0.033)	(0.031)
High	1.067	0.913^**^	0.961	0.790^***^
	(0.080)	(0.036)	(0.045)	(0.043)
**Country-level control variables**				
Log per-capita GDP	0.859	0.748^***^	1.041	0.934
	(0.137)	(0.079)	(0.184)	(0.121)
SGD_index	0.992	1.017	0.991	0.990
	(0.019)	(0.014)	(0.023)	(0.020)
Observations	37,914	38,064	38,085	38,039

p-value = ^***^p < 0.01, ^**^p < 0.05, ^*^p < 0.1.

Standard errors are reported in parentheses.

Compared to the original survey scale, the following items (i.e., Falsely Claiming Government Benefits; Cheating on tax; Accepting a bribe; Avoiding a fare on public transport) have been recoded (reversed) to allow easier interpretation of the results.

Results from this robustness check show that for one unit of increase in life satisfaction, the likelihood that individuals do not justify false state benefits increases by 9%, reject tax cheating by 10%, do not justify bribery by 11%, and refuse to cheat on public transport 8% (Table 3, models 1, 2, 3, 4). These findings are consistent with those of Ciziceno and Pizzuto (2022), who have shown that life satisfaction fosters tax morale and civic values, using a similar set variable from the World Values Survey.

## 6. Discussion

This study provides preliminary evidence that environmentalism and civicness share similar features. Both concepts seem to be moved by the same mechanism, people's wellbeing. The social capital theory (see Putnam, 1993, 2000) could explain how these associations run. Acting environmentally is a social practice expressing altruism and civic engagement, as previous studies have established (Macias, 2016). On the other side, peoples' wellbeing (i.e., life satisfaction) systematically predisposes individuals to altruism, empathy, and solidarity. Thus, individuals who are more satisfied with their life meet civic values, including preserving public goods and respecting the environment. Brown and Kasser (2005) have found that happier people live in more ecological ways than unhappy peers. Indeed, they are more focused on their community's wellbeing and may voluntarily limit their ecological footprint to benefit future generations. According to Aknin et al. (2018), a positive feedback loop exists between happiness and civic behaviors, such as donating blood or helping others. Studies discussed in previous sections, especially that of Tov and Diener (2009), indicate that subjective wellbeing increases the spirit of collaboration among citizens (i.e., increases social capital). Kushlev et al. (2020), using data from the Gallup World Poll (GWP), found that both life satisfaction and happiness were mainly associated with altruism worldwide.

Another relevant finding is that peoples' ecological attitudes differ across nations. Inglehart (1995) supports the thesis that environmental protection is higher in richer countries than in poor or underdeveloped ones and results from this study are consistent with it. According to postmaterialist theory (Inglehart, 1995, 1997), people living in economically developed countries perceive the environment as a national priority because, in those countries, postmaterialist values are prevalent. However, comparative studies have demonstrated that environmentalism is also culturally dependent. For example, Eom et al. (2016) have compared two groups of individuals from individualistic (i.e., U.S.) and collectivistic (i.e., Japan) cultures, demonstrating that cultural dimensions drive sustainable behaviors. Similarly, Xiang et al. (2019) found that individualist/collectivist orientations influence individuals' climate change actions, with collectivist people more sensitive to climate change actions.

Despite literature indicating that being connected with nature improves peoples' wellbeing [see the concept of *biophilia* in Wilson (1984)], results from the current study do not exclude diverse causal evidence. For example, Kasser (2017) finds that a reverse causal direction of the relationship between SWB and environmentalism could coexist. Following Kasser's finding, Zelenski and Desrochers (2021) have demonstrated that positive and self-transcendent emotions (including happiness) foster prosocial and pro-environmental behaviors.

## 7. Conclusion

The ecosystems' degradation has increased at alarming levels, and it is even more evident the direct repercussions of human actions on the ecological sphere. This circumstance has imposed a reflection on peoples' habits and lifestyles (York et al., 2003). Considering that actual environmental concerns (e.g., heatwaves, weather extremes, loss of biodiversity, desertification) are direct consequences of anthropic activities (IPCC, 2022), adopting pro-environmental practices could mitigate these effects. However, a better understating of which mechanisms promote individuals' environmental intentions is the prerequisite for an effective transformation of people's lifestyles. This paper offers a preliminary indication that more satisfied people meet ecocentric values and beliefs. This finding suggests that increasing the SWB levels is not only a desirable policy goal by itself. Supporting the SWB growth may offer a fertile ground for the promotion of ecological awareness and the development of more sustainable societies. There is evidence that most pro-environmental behaviors are also prosocial since they require cooperation and civic duty. It is well-noted that one of the crucial elements in implementing environmental public policies is community involvement and commitment. Thompson et al. (2011) used the expression “ecological solidarity,” indicating a conceptual way for rethinking ecological and social interdependence. Results from this study support the idea that greater life satisfaction encourages peoples' ecocentric values. In this context, the path toward the development of individuals' greener orientation involves their levels of wellbeing. However, promoting environmental matters at the individual level is not enough to change citizens' lifestyles. Policymakers should implement collective policy strategies to enhance the level of civic cooperation and people's wellbeing. As an indirect result, this could make more accessible the implementation of environmental policies aimed at modifying people's lifestyles in favor of greener social practices.

## 8. Limitations of the study and directions for future research

Some limitations affect this research. First, it is based on a representative sample from European countries that limit cross-cultural comparisons with other areas of the world. Further research in this direction could include larger samples from worldwide. Second, a potential limitation of the current research may be the nature of the data analyzed. Indeed, they came from the wave 2017–2022 of the EVS, and they do not consider the evolution of environmental values over time. Given that environmental values and individual life satisfaction are time-sensitive variables, future research on this topic should start from longitudinal data analysis.

## Data availability statement

Publicly available datasets were analyzed in this study. This data can be found here: https://doi.org/10.4232/1.13897.

## Author contributions

MC: conception and design of the research, theoretical framework, statistical analysis and interpretation of results, writing, and revising of the manuscript.

## Footnotes

^1^See for example, the School Strike for Climate (SS4C)/Fridays for Future youth collectivities: https://fridaysforfuture.org/.

^2^The VBN Theory is an applied version of the moral norm-activation theory (Schwartz, 1977) in the environmental context.

^3^In this paper, pro-environmental attitudes are considered as individuals' dispositions in respect of the environment and not their effective behavior. The concept of attitude can be expressed as the potential behavior that an individual is expected to exhibit towards a situation, event, or phenomenon (see the Ajzen and Fishbein's, 1975 model).

^4^EVS (2022).

^5^The income variable has been recoded for each country according to its distribution into three categories, each containing approximately one-third of the country's sample. For detailed information, see EVS (2022) Variable Report App. B1: mapping of income deciles into income terciles.

^6^Data on per capita GDP came from the IMF's World Economic Outlook (WEO) and are expressed in current prices (U.S. dollars per capita).

^7^Data are available at: https://eu-dashboards.sdgindex.org/downloads. For details about the Sustainable Development Goals Index see: https://eu-dashboards.sdgindex.org/downloads.

^8^However, literature alert about the so-called “social desirability bias” present in survey data (Nederhof, 1985). In some circumstances respondents give the most socially acceptable answer than their real point of view. Moreover, social desirability bias tends to be country and cultural dependent. According to McCulloch et al. (2021) giving respondents the possibility to refuse or shift the questions (as in the EVS survey) alleviates the social desirability bias.

^9^Consolidated literature shows that ignoring the ordinality of the life satisfaction data does not affect the results (Ferrer-i-Carbonell and Frijters, 2004).
